# A Rare Nonsense Mutation in the *ABCB4* Gene Associated with Progressive Familial Intrahepatic Cholestasis Type 3: A Case Report

**DOI:** 10.3390/jcm15020412

**Published:** 2026-01-06

**Authors:** Binru Cai, Duoduo Lv, Xuefeng Luo, Lingyun Zhou

**Affiliations:** 1 Center of Infectious Diseases, West China Hospital of Sichuan University, Chengdu 610041, China; caibinru@stu.scu.edu.cn (B.C.); lvduoduo@scu.edu.cn (D.L.); 2 Department of Gastroenterology and Hepatology, West China Hospital, Sichuan University, Guoxue Lane, Chengdu 610041, China; luo_xuefeng@wchscu.cn

**Keywords:** liver cirrhosis, next-generation sequencing, progressive familial intrahepatic cholestasis type 3 (PFIC3), ATP-Binding Cassette Subfamily B Member 4 (*ABCB4*), multidrug resistance protein 3 (MDR3)

## Abstract

**Background:** Progressive familial intrahepatic cholestasis (PFIC) describes a group of genetically heterogeneous disorders. Several mutations in the ATP-Binding Cassette Subfamily B Member 4 (*ABCB4*) gene have been confirmed to cause reduced phosphatidylcholine levels in bile, leading to a deficiency of biliary vesicles and instability of mixed in micelles. The disease spectrum ranges from PFIC type 3 (PFIC3) to milder conditions. Herein, we present a rare case of PFIC3 in a young woman, emphasizing the importance of early detection and management. **Methods:** The patient was diagnosed using next-generation sequencing, with genetic testing and analysis performed by the Chengdu Hua Chuang Testing Institute. Variant pathogenicity was evaluated according to the American College of Medical Genetics and Genomics guidelines and classified into five categories: pathogenic, likely pathogenic, uncertain significance, likely benign, and benign. Nomenclature was assigned following the Human Genome Variation Society standards. **Results:** Contrast-enhanced abdominal computed tomography demonstrated liver cirrhosis with marked splenomegaly. Histological examination of liver biopsy specimens using hematoxylin and eosin and Masson staining further confirmed cirrhotic changes. Genetic testing was subsequently performed and revealed a likely pathogenic variant, c.2757T > A (p. Tyr919Ter), in exon 22 of the *ABCB4* gene, which was also detected in the patient’s mother but absent in her father. Finally, PFIC3 was diagnosed. Following initiation of ursodeoxycholic acid therapy, the patient showed moderate improvement in liver function tests, underscoring a clinical case with therapeutic implications. **Conclusions:** Molecular genetic analyses of *ABCB4* are essential for the accurate diagnosis of PFIC3. Clinicians should consider cholestatic liver diseases, particularly PFIC, as a differential diagnosis in cases of liver cirrhosis with unknown etiology, especially in young patients who lack prior symptoms or a family history of liver disease.

## 1. Introduction

Progressive familial intrahepatic cholestasis (PFIC) comprises a group of rare inherited cholestatic liver disorders caused by defects in genes involved in bile formation and secretion [1,2]. To date, thirteen PFIC subtypes have been described in the Online Mendelian Inheritance in Man (OMIM) database, each associated with distinct genetic etiologies and clinical phenotypes [2]. Among them, PFIC3 results from mutations in the *ABCB4* gene, which encodes multidrug resistance protein 3 (MDR3/ABCB4), leading to impaired MDR3 function [3]. It typically presents in older children and is characterized by elevated levels of gamma-glutamyl transpeptidase (GGT) [4]. Loss of or reduction in MDR3 function decreases biliary phosphatidylcholine (PC) levels, resulting in unstable mixed micelles and increased detergent toxicity of bile salts [5]. This process progressively damages hepatocytes and biliary epithelial cells [6], ultimately causing chronic cholestasis, fibrosis, and cirrhosis. Although the diagnosis of PFIC traditionally relies on clinical features, family history, liver biopsy, and imaging, next-generation sequencing (NGS) is now considered the gold standard for genetic confirmation [7]. Ursodeoxycholic acid (UDCA) has been shown to improve liver function and clinical symptoms in various cholestatic disorders, including PFIC [8,9].

In this report, we describe a female patient with early-onset disease presenting with clinical features consistent with PFIC3, including elevated bilirubin, liver enzymes, and GGT, as well as cirrhosis, splenomegaly, and gastroesophageal varices. Genetic analysis using NGS identified a nonsense mutation c.2757T > A (p. Tyr919Ter) in exon 22 of the *ABCB4* gene, classified as “likely pathogenic” according to the American College of Medical Genetics and Genomics (ACMG) guidelines. The variant is extremely rare in population databases, with an overall allele frequency of 6.2 × 10^−7^ in the gnomAD (v4.1.0) database and absent in the East Asian population. Over an eight-year follow-up, two liver biopsies indicated disease progression, emphasizing the need for early recognition and long-term monitoring. This case highlights the importance of genetic testing and new techniques in confirming PFIC3, particularly in early-onset cases. It also outlines the current and emerging therapeutic strategies.

## 2. Methods

### 2.1. Genetic Analysis

Genomic DNA was isolated from peripheral blood collected from the proband. NGS was performed by Chengdu Huachuang Testing Institute using a clinically validated gene panel covering genes associated with inherited cholestatic liver diseases. Variant nomenclature followed Human Genome Variation Society (HGVS) guidelines. Variant pathogenicity was assessed according to the ACMG criteria. Segregation analysis was conducted by Sanger sequencing in available family members.

### 2.2. Histopathological Examination

Liver biopsy samples obtained in 2018 and 2025 were preserved in 10% neutral-buffered formalin, embedded in paraffin, and cut into 4 μm sections. Routine histopathological evaluation was performed using hematoxylin and eosin (H&E) and Masson staining. Histological features were assessed by experienced pathologists, focusing on cholestasis, inflammatory infiltration, fibrosis, and architectural changes.

### 2.3. Structural Visualization

Structural visualization of the MDR3 protein was performed using available published structural information and AlphaFold3-predicted models. These models were used solely for illustrative purposes to demonstrate the relative position of previously reported functionally important residues (V985, H989, and A990) [10] within the full-length MDR3 protein. No experimental structural validation or functional assays were performed.

## 3. Case Report

In 2018, a 17-year-old woman was incidentally found to have abnormal liver function during a routine school-related medical examination. She presented to a local hospital, where laboratory tests revealed elevated levels of bilirubin, transaminases, GGT, alkaline phosphatase, and bile acids. Abdominal ultrasonography showed diffuse parenchymal changes in the liver with splenomegaly, raising suspicion of cirrhosis. Abdominal computed tomography angiography (CTA) demonstrated reduced liver volume, splenomegaly, and prominent esophagogastric varices, consistent with portal hypertension and cirrhosis. Liver biopsy in 2018 revealed partial disruption of the lobular architecture. Hepatocellular hydropic degeneration and focal spotty necrosis were observed, along with intracellular cholestatic pigment deposition. The portal tracts were expanded with lymphocytic infiltration. Cholestasis was present within hepatocytes, accompanied by hepatic fibrosis. Liver stiffness measurement was 14.2 kPa. Secondary causes of liver dysfunction and cirrhosis, including various forms of viral hepatitis, autoimmune hepatitis, and IgG4-related liver disease, were all excluded. As the etiology of the liver cirrhosis remained unclear, the patient initially received symptomatic treatment. She was regularly followed up at the hospital, with persistent findings of cirrhosis and splenomegaly. The patient received UDCA therapy for several months, which was subsequently discontinued. Liver function tests remained abnormal, and a gradual decline in platelet counts was observed during follow-up.

**Figure 1:**
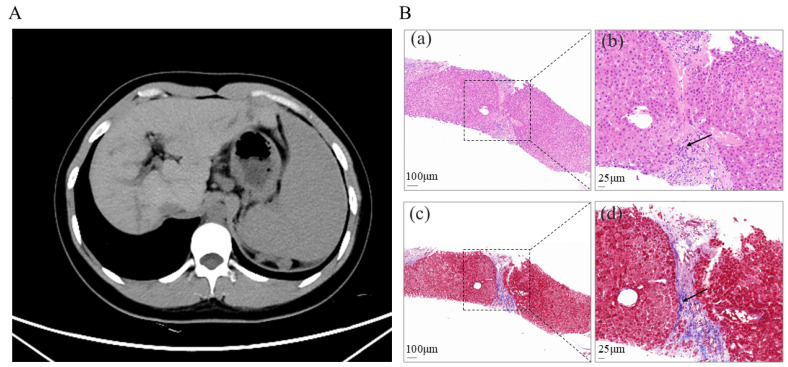
(**A**). Abdominal enhanced CT image showing liver cirrhosis with significant splenomegaly. (**B**). Histological section of liver biopsy showing cirrhosis changes, confirmed by H&E and Masson staining. (**a**). Portal tract (dashed box), H&E staining. Scale bar = 100 μm. (**b**). High-magnification view of the dashed boxed area in (**a**), showing fibrous tissue (arrows), H&E staining. Scale bar = 25 μm. (**c**). Portal tract (dashed box), Masson staining. Scale bar = 100 μm. (**d**). High-magnification view of the dashed boxed area in (**c**), showing fibrous tissue (arrows), Masson staining. Scale bar = 25 μm.

**Figure 2:**
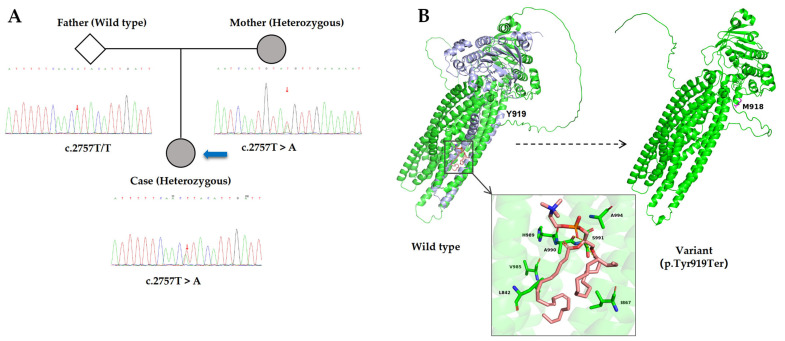
(**A**). Sanger sequencing chromatograms of exon 22 of the *ABCB4* gene for the father, mother, and proband. The three small red arrows indicate the position of nucleotide c.2757 in these individuals. The large blue arrow points to the proband. The father exhibits a homozygous wild-type sequence (T/T), while both the mother and the proband show a heterozygous T > A substitution (c.2757T > A), confirming maternal inheritance of the variant. (**B**). AlphaFold3-predicted models show the full-length wild-type MDR3 (left) and the truncated MDR3 protein caused by the p. Tyr919Ter mutation (right). In the wild-type structure, the purple-blue region indicates the C-terminal segment downstream of Tyr919, which is absent in the truncated variant. The inset highlights residues V985, H989, and A990, previously reported to be critical for PC binding and translocation. Models were used solely for visualization of relative residue positions, without functional or experimental validation.

To further investigate the underlying etiology and assess the current hepatic status, the patient was hospitalized at our institution on 7 April 2025. Contrast-enhanced computed tomography (CT) of the abdomen revealed findings consistent with cirrhosis, splenomegaly, and formation of portosystemic collateral circulation (Figure 1A). The liver stiffness measurement was 15.8 kPa. Liver biopsy in 2025 demonstrated hepatocellular regeneration, with scattered focal spotty necrosis within the lobules and mild piecemeal necrosis at the limiting plate. Portal tract infiltration by lymphocytes, monocytes, occasional plasma cells, and neutrophils was observed. Masson staining revealed fibrous tissue proliferation and expansion of the portal tracts, with visible fibrous septa and pseudo-lobule formation. These findings were consistent with nodular cirrhosis, corresponding to histological grading and staging of G2S4 (Figure 1B), indicating disease progression. Genetic analysis using NGS revealed a heterozygous nonsense variant in *ABCB4* (NM_000443.4): c.2757T > A (p. Tyr919Ter) in exon 22. This variant introduces a premature termination codon at amino acid position 919. Segregation analysis within the family showed that the variant was maternally inherited and was absent in the father (Figure 2A).

Based on the patient’s clinical phenotype and genetic findings, this case represents PFIC3 caused by an *ABCB4* mutation. According to the ACMG guidelines [11], this variant is classified as “likely pathogenic” based on the following evidence: (1) PVS1: This is a nonsense variant predicted to result in loss of function, annotated as “stop gained” with a high-confidence protein loss-of-function (pLoF) prediction by Ensembl VEP. (2) PM2: The variant is extremely rare in population databases, with an overall allele frequency of 6.2 × 10^−7^ in gnomAD (v4.1.0) and is absent in the East Asian subpopulation. (3) PP3: Multiple in silico prediction tools consistently support a deleterious effect, as exemplified by a very high Combined Annotation Dependent Depletion (CADD) score of 37.0, which is strongly associated with harmful effects. (4) PP4: The phenotype of this case, early-onset cholestasis with markedly elevated GGT, is highly suggestive of PFIC3 caused by *ABCB4* mutations.

Furthermore, Jeppe A. Olsen et al. [10] identified three critical residues—V985, H989, and A990—that are essential for PC binding and translocation by MDR3. The p. Tyr919Ter nonsense mutation is located upstream of these residues, resulting in premature termination of the MDR3 protein before the region encompassing these functionally important sites. Structural visualization based on available structural information for MDR3- and AlphaFold3-predicted models was used to illustrate the relative position of these critical residues within the full-length protein (Figure 2B). In the wild-type MDR3 structure, the region shown in purple blue represents the C-terminal portion of the protein synthesized downstream of Tyr919. No functional assays or experimental structural validation were performed in this study. Taken together, the truncation occurring upstream of these essential residues supports a pathogenic mechanism involving impaired MDR3-mediated PC transport, consistent with the established molecular basis of PFIC3.

**Figure 3:**
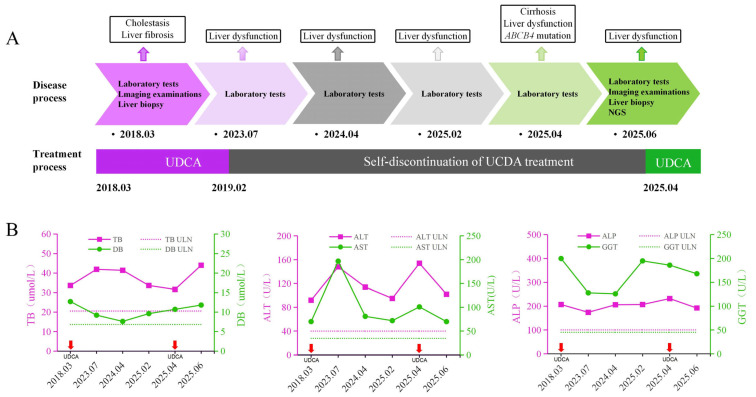
Dynamic changes in liver function test results and treatment process. (**A**). Timeline of disease progression and treatment. (**B**). Dynamic changes in liver function by laboratory tests. The x-axis represents follow-up time points. In each panel, solid magenta and green lines denote the patient’s liver biochemical parameters, with the corresponding values shown on the left and right y-axes, respectively. Horizontal dashed lines indicate the upper limits of normal (ULN). The short red arrow indicates the time point at which oral UDCA intervention treatment was given. NGS, next-generation sequencing; UDCA, ursodeoxycholic acid; TB, total bilirubin; DB, direct bilirubin; ALT, alanine aminotransferase; AST, aspartate aminotransferase; ALP, alkaline phosphatase; GGT, gamma-glutamyl transferase; ULN, upper limit(s) of normal.

Two liver biopsies, performed in 2018 and again in 2025, demonstrated disease progression from intrahepatic cholestasis with fibrosis to nodular cirrhosis, accompanied by inflammatory infiltration and bile duct hyperplasia. Although initially prescribed in 2018, the patient had discontinued UDCA therapy prematurely. After resuming UDCA treatment following hospitalization in April 2025, the patient experienced modest biochemical improvement in liver function test, with a reduction in liver enzyme levels within two months (Figure 3).

## 4. Discussion

Defining the genetic basis of cholestatic disorders is essential for advancing our understanding of liver disease pathogenesis and for guiding the development of more precise diagnostic and therapeutic approaches. In this study, we report a rare nonsense mutation c.2757T > A (p. Tyr919Ter) of the *ABCB4* gene in a patient whose clinical features were consistent with a diagnosis of PFIC3. The p. Tyr919Ter mutation introduces a premature stop codon upstream of the critical MDR3 residues V985, H989, and A990 required for PC transport [10]. Structural visualization based on available MDR3- and AlphaFold3-predicted models illustrates the loss of this functionally essential C-terminal region. This truncation supports a pathogenic mechanism involving impaired MDR3-mediated PC transport, consistent with PFIC3.

Furthermore, this mutation introduces a premature termination codon, leading to the production of a truncated MDR3 protein. Although the truncated protein may still be expressed, it is likely to be poorly detected due to rapid degradation via the endoplasmic reticulum-associated degradation system [12,13,14]. This rapid degradation prevents MDR3 from performing its normal function, such as PC binding and translocation, which are essential for bile acid transport [15]. Experimental validation, such as immunofluorescence in cell models or immunohistochemistry in patient biopsies, could confirm the presence and degradation of this truncated protein. Despite substantial progress in genetic testing, challenges persist. Phenotype–genotype correlations are not always consistent [16], making the interpretation of molecular findings a continually evolving process.

PFIC3 is caused by a genetic mutation that leads to the production of an impaired MDR3 protein, disrupting its normal function. UDCA therapy has shown benefits only in patients with residual MDR3 protein activity. For example, a PFIC3 patient with residual MDR3 expression resolved fibrosis and cirrhosis after 9 years of UDCA treatment [17]. By contrast, three children with *ABCB4* mutations that result in complete loss of MDR3 expression/function exhibited progressive liver disease that was refractory to UDCA treatment [18]. Furthermore, patients with biliary phospholipid levels exceeding 6.9% of the total biliary lipid content were more likely to respond to UDCA treatment and exhibit prolonged native liver survival [19]. Upon the initial detection of intrahepatic cholestasis, long-term oral UDCA therapy should be considered as a symptomatic treatment to improve liver function and slow disease progression of patients with residual MDR3 activity. The recommended therapeutic dose for individuals with cholestatic liver diseases is approximately 15 mg/kg/day [20]. Liver biopsies performed seven years apart demonstrated histological progression in this case. However, after two months of UDCA treatment, liver function tests showed improvement with lower liver enzymes. Thus, although UDCA does not alter the outcome of PFIC3, it can slow down the progression of this patient if residual MDR3 function is preserved.

In addition to UDAC, several oral agents have been explored in cholestatic liver diseases and may be relevant for the management of PFIC3 [8]. Obeticholic acid, a farnesoid X receptor (FXR) agonist, reduces bile acid synthesis and hepatic bile acid accumulation and may serve as an adjunct therapeutic option to alleviate bile-acid-induced hepatobiliary injury in PFIC3 [21,22]. By contrast, ileal bile acid transporter (IBAT) inhibitors reduce hepatic bile acid burden by interrupting enterohepatic circulation. For example, odevixibat is the first approved IBAT inhibitor for the treatment of selected PFIC subtypes [23]. Furthermore, in a multicenter, randomized, double-blind, placebo-controlled phase 3 trial (NCT03905330), maralixibat, an IBAT inhibitor, significantly improved pruritus and reduced serum bile acid levels in patients with PFIC [24,25]. In addition, norUDCA (norucholic acid, NCA) exerts protective effects on cholangiocytes by stimulating bicarbonate-rich choleresis, thereby attenuating bile-acid-mediated toxicity; this mechanism is particularly evident in the *MDR2* knockout mouse model lacking biliary phospholipids [5,26]. Experimental gene-based therapeutic approaches have also been explored. For instance, delivery of synthetic human *ABCB4* mRNA via lipid nanoparticles restored liver function and halted disease progression in a PFIC3 mouse model [27]. Similarly, gene therapy using a recombinant adeno-associated virus (rAAV) vector carrying the MDR3 coding sequence (AAV8-MDR3) has shown therapeutic potential in infant PFIC3 mouse models [28].

Liver transplantation remains the only curative option for PFIC3, offering improvement in cholestasis and clinical symptoms in 75–100% of patients over a short-term follow-up period of 3–5 years [29]. Early-onset PFIC3 significantly affects children’s growth and overall health; however, advances in pediatric liver transplantation have offered new prospects for children with end-stage liver disease [30]. Despite this, the limited availability of donor livers and the lifelong need for immunosuppressive therapy pose significant challenges to this treatment. Therefore, the development of more effective pharmacological therapies is urgently needed to slow the progression of cirrhosis and enhance patient outcomes and quality of life.

## 5. Conclusions

PFIC3 is an autosomal recessive disease that results from mutations in the *ABCB4* gene. Current diagnostic approaches for PFIC3 involve clinical, biochemical, imaging, and histological evaluations. However, definitive diagnosis depends on genetic testing. Initial PFIC3 management emphasizes nutritional support and symptomatic relief. However, most patients eventually need liver transplantation due to progressive liver fibrosis and cirrhosis. In young patients with cirrhosis of unknown etiology, particularly in the absence of prior symptoms or a family history of liver disease, cholestatic liver disorders, including PFIC, should be considered to allow timely diagnosis and appropriate management.

## Data Availability

The original contributions presented in this study are included in the article. Further inquiries can be directed to the corresponding author.

## Abbreviations

The following abbreviations are used in this manuscript:

PFIC	Progressive familial intrahepatic cholestasis
*ABCB4*	ATP-binding cassette subfamily B member 4
OMIM	The online mendelian inheritance in man
MDR3	Multidrug resistance protein 3
GGT	Gamma-glutamyl transferase
PC	Phosphatidylcholine
NGS	Next-generation sequencing
UDCA	Ursodeoxycholic acid
gnomAD	Genome Aggregation Database
HGVS	The Human Genome Variation Society
ACMG	The American College of Medical Genetics and Genomics
H&E	Hematoxylin and eosin
CTA	Computed tomography angiography
CT	Computed tomography
CADD	Combined annotation dependent depletion
ULN	Upper limit(s) of normal
TB	Total bilirubin
DB	Direct bilirubin
ALT	Alanine aminotransferase
AST	Aspartate aminotransferase
ALP	Alkaline phosphatase
FXR	Farnesoid X receptor
IBAT	Ileal bile acid transporter
NCA	Norucholic acid
*MDR2*	Multidrug resistance protein 2
rAAV	Recombinant adeno-associated virus
